# Enhancing patient‐specific quality assurance in MR‐guided radiation therapy: A fluence‐based method using log files

**DOI:** 10.1002/acm2.70238

**Published:** 2025-09-03

**Authors:** Kai Yuan, Matthew Man‐hin Cheung, Louis Lee

**Affiliations:** ^1^ Medical Physics Division Department of Medical Innovation & Technology CUHK Medical Centre Hong Kong SAR China; ^2^ Department of Clinical Oncology Faculty of Medicine The Chinese University of Hong Kong Hong Kong SAR China

**Keywords:** gamma analysis, log file, MR‐Linac, patient‐specific quality assurance, radiation therapy

## Abstract

**Background:**

Patient‐specific quality assurance (PSQA) is crucial in radiation therapy to ensure accurate and safe dose delivery. The Elekta Unity MR‐Linac system, which combines MRI with a linear accelerator, presents unique challenges for conventional PSQA methods due to its adaptive capabilities and the presence of a magnetic field.

**Purpose:**

This study introduced a novel PSQA method for the Elekta Unity MR‐Linac system, utilizing treatment log files and fluence map verification to provide a more efficient alternative to traditional measurement‐based techniques.

**Methods:**

The proposed method analyzed log file data, including monitor units (MU), multi‐leaf collimator (MLC) positions, and jaw positions, comparing them with treatment planning system (TPS) values. Fluence maps were generated for each gantry angle and evaluated using gamma analysis. A user‐friendly interface was developed to streamline the process.

**Results:**

The method was tested on nine stereotactic body radiation therapy (SBRT) cases, showing strong concordance between planned and delivered parameters. MU deviations were within 0.5 MU, X‐jaw and MLC leaf position deviations were under 1 mm, and gamma analysis of fluence maps achieved passing rates above 98% for all gantry angles.

**Conclusion:**

This log file‐based PSQA method offered distinct advantages over traditional measurement‐based approaches, including reduced QA time, direct assessment of the delivered plan, and comprehensive evaluation of treatment parameters. This method provided an efficient and accurate alternative PSQA solution for MR‐guided adaptive radiotherapy.

## 1 INTRODUCTION

Patient‐specific quality assurance (PSQA) is a critical process that checks the accuracy and safety in the radiation dose delivery.^1^ PSQA verifies whether the delivered dose matches with the planned dose in the target and surrounding normal tissues. PSQA methods can be broadly categorized into two main types: measurement‐based QA and calculation‐based QA.^2^, ^3^ Measurement‐based QA employs physical measurements to verify dose delivery accuracy, utilizing tools such as diode arrays, ionization chambers, Electronic Portal Imaging Devices (EPID),^4^ and radiochromic films.^5^ In contrast, calculation‐based QA does not require actual dose delivery. Instead, it relies on independent dose calculations using a separate dose calculation engine or theoretical checks on the treatment plan.^3^

The Elekta Unity MR‐Linac machine (Elekta AB, Stockholm, Sweden) is a cutting‐edge radiation therapy system that combines a 1.5‐Tesla magnetic resonance imaging (MRI) scanner with a 7 MV flattening filter‐free linear accelerator (LINAC).^6^ This innovative technology allows for adaptive treatment plans that can adjust to daily changes in tumor shape and position. The online adaptive capabilities of the Unity system, including adaptive‐to‐shape (ATS) and adaptive‐to‐position (ATP), necessitate a new plan for each treatment fraction.^7^ Consequently, PSQA is required for every Unity treatment session. However, conventional measurement‐based QA is impractical, as the patient is already positioned on the couch after online adaptation. Furthermore, the presence of a magnetic field affects electron trajectories, requiring TPS to account for this effect in dose calculations. Monte Carlo simulation is the most accurate method for modeling magnetic field effects, but its computational intensity makes it unsuitable for pre‐treatment PSQA.^8^ In practice, our center performs a rapid independent calculation of the dose at the center of planning target volume immediately in RadCalc (LifeLine Software, Austin, Texas, USA)^9^ after online adaptation for both ATP and ATS plans to detect significant errors. After the treatment, the adapted plan is delivered to an ArcCheck phantom^10^, ^11^ for comprehensive PSQA.

Log files record detailed information during actual dose delivery, including MU, gantry angle, MLC position, and jaw position. There is growing interest in replacing traditional measurement‐based quality assurance with delivery log file analysis for PSQA. Several other studies have also investigated the feasibility of adopting log file to perform PSQA in conventional Linacs and demonstrated promising results.^12^, ^13^, ^14^ This study proposes using log files for PSQA of Elekta Unity plans, focusing on fluence map verification for each gantry angle. Gamma analysis is employed to compare fluence distributions between the log file and the TPS plan, providing a comprehensive and efficient method for ensuring treatment accuracy.

## 2 METHODS AND MATERIALS

The Elekta Unity system features a source‐axis distance (SAD) of 143.5 cm and a maximum field size of 57.4 cm (IEC X) × 22.0 cm (IEC Y). The X jaws move across the cross‐plane direction, while the 160 MLC leaves move in the in‐plane direction (Y direction). The log files, sampled at every 40 ms, were exported from rich text format (.rtf) to comma‐separated values (.csv) files in Elekta's proprietary converter, enabling further analysis using in‐house MATLAB (MathWorks, Natick, Massachusetts, USA) scripts. In certain cases, treatment plans were divided into two or more sub‐plans due to limitations on the number of segments allowed per sub‐plan. Additionally, radiation delivery might be manually interrupted by therapists in response to unforeseen circumstances. These scenarios resulted in the generation of multiple log files. To ensure a comprehensive analysis, information from these separate files was systematically organized and merged into a single consolidated file.

**FIGURE 1:**
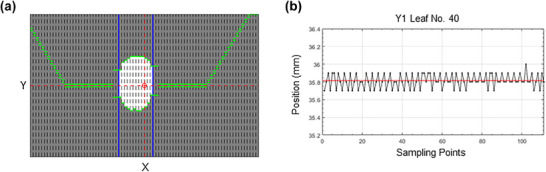
(a) Geometry of the selected segment. The red dashed lines indicate the machine isocenter. Green lines represent MLC positions, and blue lines represent the X jaws. The dark area indicates the region blocked by MLCs or jaws, while the white area represents the open field. Black dashed lines define the edges of each MLC leaf pair. (b) Leaf position data from the optical leaf tracking system for Y1 leaf No. 40. The nominal leaf position was 36 mm, whereas the actual leaf position recorded in the log file was 35.81 ± 0.08 mm. The red solid line represents the average leaf position.

Each treatment plan in the Elekta Unity system operated under three distinct Linac states: “Intersegment,” “Move Only,” and “Radiation On,” with radiation beams generated exclusively during the “Radiation On” state. The step‐and‐shoot delivery mode facilitated easy segment separation based on the Linac State variable. MU delivered per segment were recorded in the Step Dose variable, which reset to zero upon beam deactivation for each segment. MLC positions (Y1 and Y2) and jaw positions (X1 and X2) were stored as scaled values relative to the isocenter plane, measured in millimeters. To reduce the effect of inherent fluctuations in Unity's optical tracking system, the positions of MLCs and jaws were averaged across all sampling points within each segment, providing more reliable positional data for analysis. To demonstrate the inherent variability of MLC and jaw positions recorded in the log file, we selected an example from a single control point. This segment delivered 17.2 MU across 111 sampling points. The geometry of this segment is illustrated in Figure 1a, while the leaf position data from the optical leaf tracking system for Y1 leaf No. 40 is shown in Figure 1b. The nominal leaf position is 36 mm, whereas the actual leaf position recorded in the log file is 35.81 ± 0.08 mm.

Comparative analyses were conducted between the TPS plan and the log file for nine SBRT plan, focusing on MU, X‐jaw positions, and MLC settings. For MU and X‐jaw positions, deviations were calculated by subtracting log file values from TPS plan values for corresponding segments. MLC position analysis involved identifying the maximum error across all segments for each MLC leaf and computing the root mean square (RMS) error across segments. The RMS error calculation provided a quantitative measure of the overall discrepancy between planned and delivered MLC positions. The formula used for the RMS error calculation is as follows:

$$Y_{RMS,i}=\sqrt{\frac{1}{m}{\sum_{j=1}^{m}\left(Y_{log,i}\;-\;Y_{plan,i}\right)}^{2}}$$

m is the number of segments in the plan, $Y_{RMS,i}$ is the RMS for $i^{th}$ MLC.

For each gantry angle, a fluence map at the isocenter plane was calculated by summing the fluence contributions from individual segments, weighted by their respective MU. The isocenter is defined at the center of the symmetric field shaped by the jaws and MLCs, positioned at the plane corresponding to the SAD of 143.5 cm. This approach provided a comprehensive representation of the radiation delivery pattern. To facilitate a thorough comparison between the TPS and log file‐derived fluences, separate fluence maps were computed for all gantry angles. Each segment was defined as a 2D binary mask shaped by MLCs and jaws. The calculation can be expressed mathematically as:

$$F_{\log,i}=\sum_{j=1}^{n}MU_{j}\times segment_{j}$$

where j is the index for the n segments in gantry angle i.

The fluence maps derived from the TPS plan and the log file were compared using gamma analysis, a method that evaluated the displacement between corresponding fluence distributions. This analysis was performed independently for each point in the fluence map, with a resolution of 0.1 mm. The gamma index quantified the agreement between two points, $\;\vec{r_{plan}}$ and $\vec{r_{log}}$, representing positions in the planned and delivered fluence distributions, respectively. This approach provided a comprehensive assessment of the spatial and dosimetric accuracy of the treatment delivery, allowing for the identification of any discrepancies between the intended and actual radiation patterns. The calculation can be expressed mathematically as ^2^:

$$\Gamma \left(\vec{r_{plan}},\vec{r_{log}}\right)=\sqrt{\frac{r^{2}\left(\vec{r_{plan}},\vec{r_{log}}\right)}{\Delta d^{2}}+\frac{\delta^{2}\left(\vec{r_{plan}},\vec{r_{log}}\right)}{\Delta D^{2}}}$$

$$\gamma \;\left(\vec{r_{plan}}\right)=\min\left(\Gamma \left(\vec{r_{plan}},\vec{r_{log}}\right)\right)$$

where $r(\vec{r_{plan}},\vec{r_{log}})$ is the distance between two corresponding points, $\delta (\vec{r_{plan}},\vec{r_{log}})$ is the dose difference, Δd is the distance to agreement (DTA) criterion in mm, and ΔD is the dose difference criteria in %. In the analysis, Δd was set to 1 mm and ΔD was set to 1%. A threshold of 10% of the global maximum fluence was applied to focus the gamma analysis on clinically relevant areas. Only points with fluence values exceeding this threshold were included in the calculation. The gamma analysis results were quantified as a passing rate percentage, considering all points above the threshold. A gamma index (γ) between 0 and 1 indicated that a point has met the specified criteria, while values exceeding 1 signified a failure to meet these criteria.

Pearson's correlation was employed to identify systematic fluence shifts and served as a supplementary tool for assessing the agreement between delivered and planned fluence maps. According to a study that used Pearson's correlation to compare fluence maps derived from TPS plans and log files for volumetric‐modulated arc therapy (VMAT) SBRT plans, a threshold of 0.985 was selected for the correlation coefficient.^15^ We adopted this value as a preliminary threshold. To further validate this correlation coefficient threshold, we considered a scenario involving a systematic 1 mm shift in the fluence distribution caused by potential MLC errors. Data from all nine subjects were analyzed, pooling measurements across all gantry angles.

To enhance user experience, a custom MATLAB user interface (UI) was developed for performing gamma analysis and visualizing results. This study was carried out in accordance with the Declaration of Helsinki. This study was approved by the relevant Clinical Research Ethics Committee.

## 3 RESULTS

**FIGURE 2:**
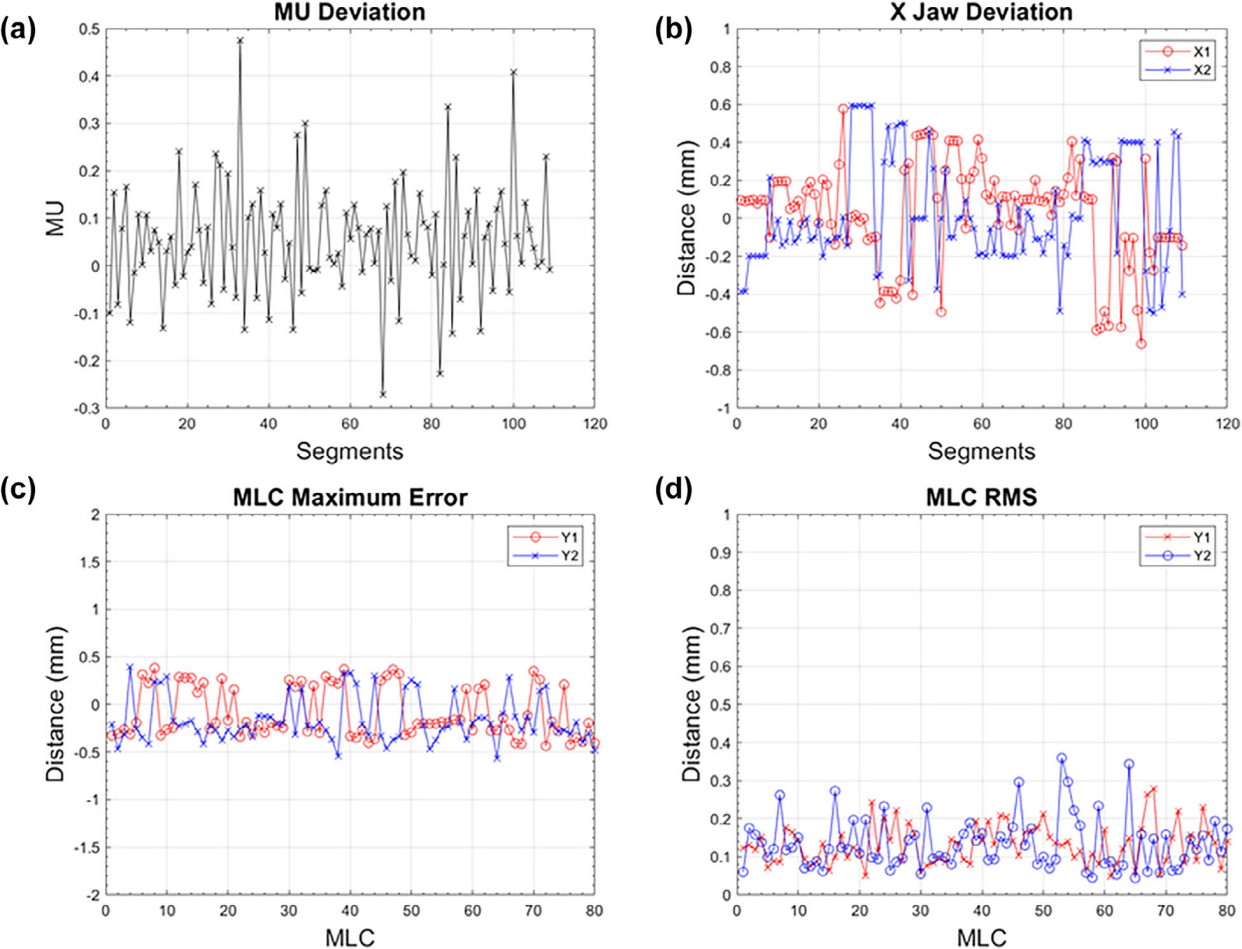
Comparison of planned versus delivered treatment parameters. (a) MU deviations between treatment plan and delivery log files. (b) X‐jaw positioning deviations (mm). (c) Maximum leaf position deviations across all segments, displayed by leaf pair. (d) RMS of leaf position deviations across all segments, displayed by leaf pair.

The details of the nine plans are presented in Table 1. All nine plans, spanning different treatment sites and varying numbers of segments, consistently achieved high gamma passing rates across all gantry angles. To illustrate the results, a prostate case with 109 segments and 15 gantry angles was selected as an example. Figure 2a demonstrates MU deviations consistently within ± 0.5 MU for all segments. X‐jaw positioning, as shown in Figure 2b, exhibits deviations less than 1 mm across all segments. Figure 2c illustrates the maximum leaf position deviation for each leaf pair across all segments, with all deviations within ± 1 mm. Figure 2d presents the RMS of leaf position errors, identifying leaves with relatively larger positional variations.

**TABLE 1:** Details for the nine SBRT plans.

Case no.	Treatment site	Number of gantry angles	Number of segments	Total MU	Gamma analysis results
1	Spine	17	135	1863.02	99.68 ± 0.13
2	Thorax	13	316	3228.71	99.68 ± 0.16
3	Brain	11	105	1375.12	99.64 ± 0.52
4	Prostate	17	346	4232.35	99.97 ± 0.02
5	Abdomen	15	226	2980.68	99.81 ± 0.16
6	Prostate	15	109	1629.49	99.85 ± 0.06
7	Prostate	17	201	2614.20	99.74 ± 0.09
8	Liver	16	290	4094.56	99.80 ± 0.07
9	Brain	13	75	1446.77	99.75 ± 0.21

**FIGURE 3:**
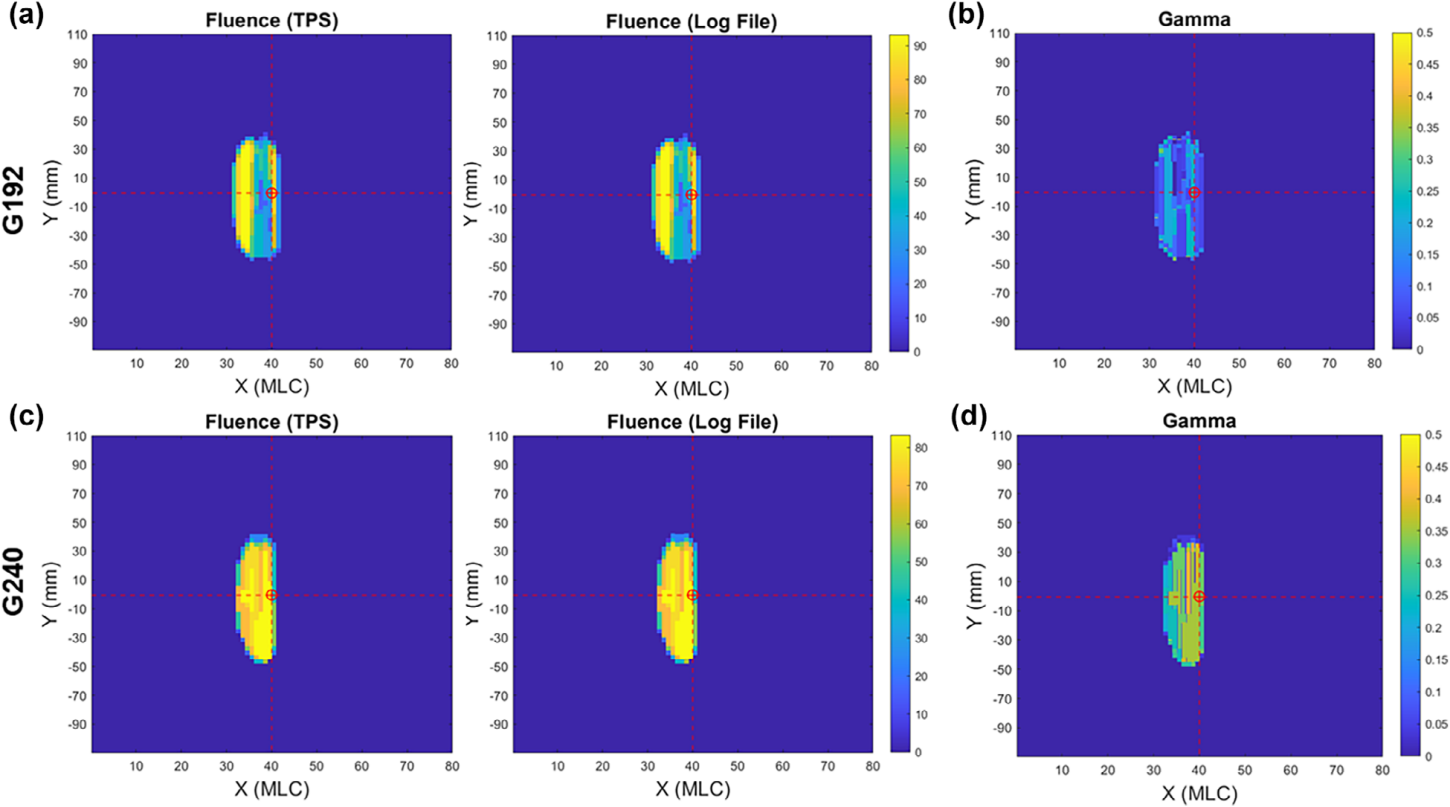
Comparison of planned and delivered fluence maps with gamma analysis. (a) The TPS fluence map and log file‐derived fluence map in MU for gantry angles 192°. The red dashed lines indicate the machine isocenter. (b) The gamma analysis results for gantry angle 192°. (c) The TPS fluence map and log file‐derived fluence map in MU for gantry angles 240°. (d) The gamma analysis results for gantry angle 240°.

Figure 3 presents a comprehensive analysis of fluence maps and gamma analysis results for two specific gantry angles. Figure 3a and c display the fluence maps for gantry angles 192° and 240°, respectively. In each figure, the left panel shows the TPS fluence map, while the right panel depicts the log file‐derived fluence map. Fluence intensities are quantified in MU. The isocenter is clearly demarcated by a red dashed line and a red circle for reference. Figure 3b andd illustrate the corresponding gamma analysis results for the 192° and 240° gantry angles. Notably, 99.77% and 99.85% of the points in the gamma analysis results fall within the 0–1 range, respectively.

**FIGURE 4:**
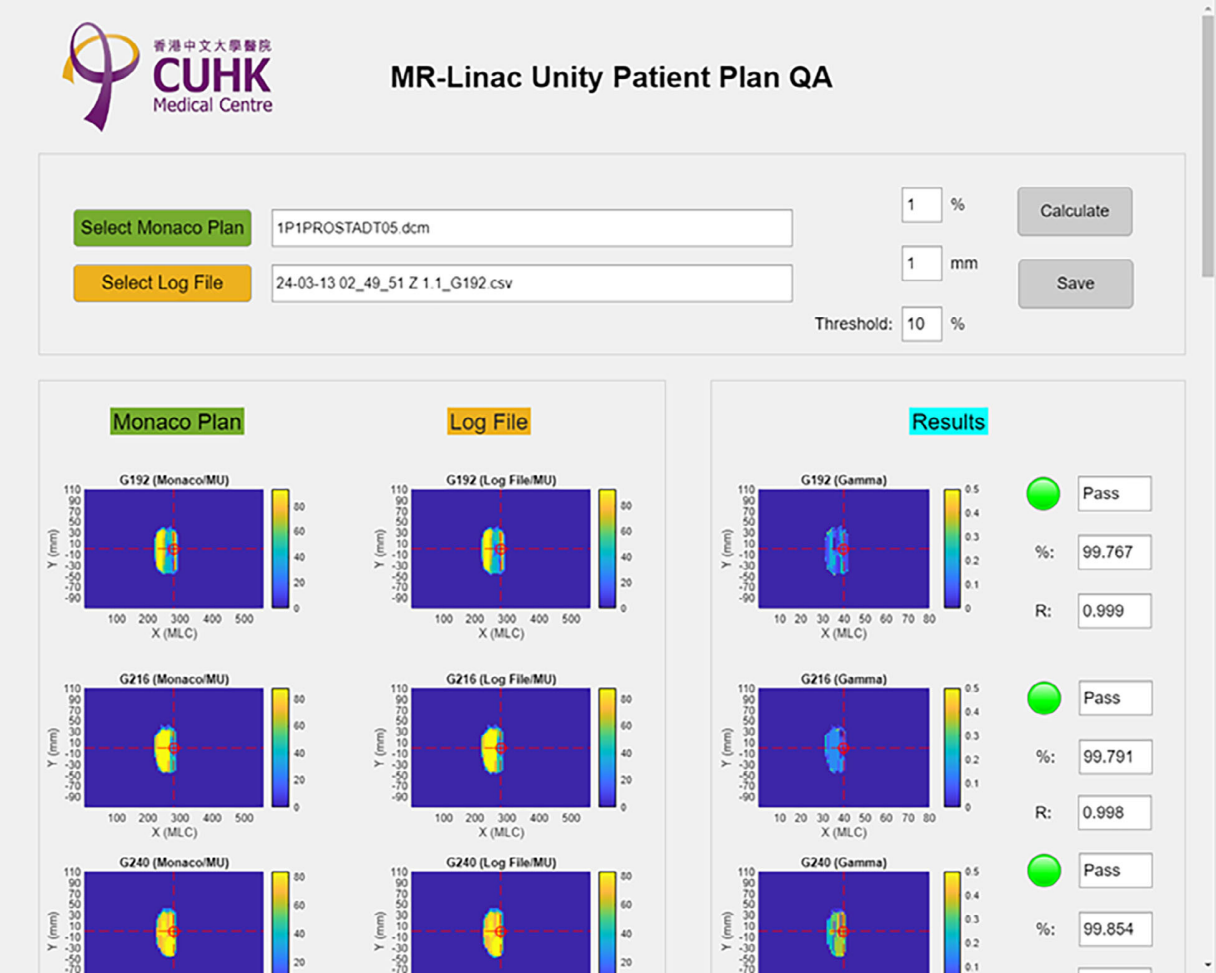
UI for log file‐based patient‐specific QA.

Figure 4 illustrates the UI developed for log file‐based patient plan QA. Users can select the TPS plan DICOM file and the corresponding log file. Gamma analysis is performed with ΔD=1% and Δd=1mm, and a 10% fluence threshold to focus on clinically relevant areas. The UI displays gamma passing rates for each gantry angle, marking rates above 98% as “PASS” for quick assessment. Pearson's correlation coefficients are also provided, offering an additional measure of agreement between planned and delivered fluence distributions. All correlation coefficients were well above the threshold. Using data with a 1 mm systematic error introduced, the calculated Pearson's correlation was 0.987 ± 0.008, with a 95% confidence interval of 0.986–0.988.

## 4 DISCUSSION

This study introduced a novel PSQA method for an MR‐Linac, utilizing treatment log files and implemented through a user‐friendly interface. The method focused on fluence map verification for each gantry angle. By directly examining the delivery parameters, this approach offered a more detailed and efficient alternative to conventional measurement‐based QA techniques.

Monaco TPS used in Elekta Unity accurately accounts for the complex effects of the magnetic field on dose distribution. It precisely models the behavior of secondary electrons, including the electron return effect, which is unique to MR‐Linac systems.^16^ Currently, Monaco TPS is the only available system capable of modeling magnetic field effects on dose distribution. Calculating radiation dose without considering the magnetic field leads to inaccurate modeling of the actual dose distribution, as demonstrated in previous studies.^8^ This limitation poses significant challenges for pre‐treatment dose calculations, particularly when the patient is already positioned on the treatment couch. The log analysis can also verify the plan transfer integrity between the TPS and the treatment console. Therefore, comprehensive PSQA is often after treatment delivery.

Log file‐based PSQA offers distinct advantages over traditional measurement‐based approaches. This method significantly reduces quality assurance time by utilizing data recorded during actual treatment delivery, eliminating the need for extensive phantom measurements. Log file analysis directly assesses the real delivered plan, avoiding potential uncertainties introduced by secondary phantom‐based measurements such as those using ArcCheck phantom.^10^, ^11^ Log files capture a comprehensive range of parameters, including MLC position, dose rate, and treatment delivery time, enabling a more thorough evaluation of the entire treatment process. This detailed analysis surpasses the capabilities of superimposed dose measurements, providing a more nuanced and accurate assessment of treatment delivery. Utilizing photon fluence as the primary indicator for plan comparison offers significant advantages in MR‐guided radiation therapy QA. This approach eliminates the need for complex dose calculations that require accurate magnetic field modeling. Gamma analysis, applied to fluence distributions, enables a point‐wise comparison between planned and delivered treatments,^2^, ^17^ providing exceptional sensitivity in detecting anomalies in MLC position or MU delivery. Pearson's correlation is effective in detecting systematic fluence shifts. Our estimated correlation coefficient threshold of 0.987, corresponding to a 1 mm systematic distribution shift, is consistent with the findings of a previous study.^15^ Furthermore, the proposed method implements a gantry‐specific analytical approach that enables rapid identification of delivery errors within treatment plans. The system provides detailed performance metrics including gantry angle verification, MU accuracy assessment, and comprehensive MLC performance evaluation. While a previous study has validated the feasibility of log file‐based PSQA for Elekta Unity using the commercial software LinacView (Standard Imaging, Madison, Wisconsin, USA),^12^ our methodology addresses the limitations of proprietary solutions. We presented a comprehensive, step‐by‐step methodology for developing an in‐house PSQA software designed for the Elekta Unity MR‐Linac platform. This log file‐based PSQA method offers broad applicability across various online adaptive treatment systems, providing institutions with a cost‐effective and customizable QA solution.

Several limitations are worthy to be mentioned. While log files provide valuable data, they may not perfectly represent actual dose delivery due to potential discrepancies between recorded and actual machine parameters. Additionally, fluence map analysis alone cannot directly assess dose distribution within patient tissue. Nevertheless, the proposed log file‐based verification approach demonstrates robust performance as an alternative to conventional post‐treatment ArcCheck measurements for adapted plans.

The upgraded Elekta Unity Comprehensive Motion Management (CMM) system introduces a sophisticated gating feature that tracks tumor motion in real‐time, automatically pausing the beam when the target moves beyond defined margins.^18^ This intermittent radiation delivery pattern makes traditional post‐treatment QA measurements impossible to accurately reproduce the discontinuous treatment process. In such scenarios, patient‐specific QA based on log files provides a more faithful representation of the actual treatment delivery. Our QA tool can be conveniently adapted to these gated treatment plans, ensuring comprehensive verification of delivery accuracy. The discontinuous MU delivery and organ motion can both be incorporated into the PSQA workflow.

## 5 CONCLUSION

The proposed log file‐based PSQA approach streamlines the QA process, allows for direct evaluation of delivered plans, and provides a thorough assessment of treatment parameters. This method offers a robust and time‐efficient solution for post‐treatment PSQA in MR‐guided adaptive radiotherapy.

## AUTHOR CONTRIBUTIONS

Kai Yuan performed the experimental investigation, data analysis, and manuscript preparation. Matthew Man‐hin Cheung contributed to the experimental work and manuscript preparation. Louis Lee conceptualized the study and provided supervision. All authors reviewed and approved the final version.

## CONFLICT OF INTEREST STATEMENT

The authors declare no conflicts of interest.

## ETHICS STATEMENT

This study was approved by the Clinical Research Ethics Committee of CUHK Medical Centre (CREC‐202505).
